# Study designs, measures and indexes used in studying the structural racism as a social determinant of health in high income countries from 2000–2022: evidence from a scoping review

**DOI:** 10.1186/s12939-022-01796-0

**Published:** 2023-01-06

**Authors:** Md Koushik Ahmed, Desiree Scretching, Sandra D. Lane

**Affiliations:** 1 Department of Public Health, Falk College of Sports and Human Dynamics, Syracuse University, 150 Crouse Dr, 430 White Hall, Syracuse, NY 13244 USA; 2 School of Information Studies, Syracuse University, 343 Hinds Hall, Syracuse, NY 13244 USA; 3 Department of Public Health, Falk College of Sports and Human Dynamics, 439 White Hall, Syracuse University, Syracuse, NY 13244 USA

**Keywords:** Structural racism, Social determinants of health, Exposure, Outcome, Indexes of measurement, Community participatory research approach

## Abstract

**Background:**

Globally, structural racism has been well documented as an important social determinant of health (SODH) resulting in racial inequality related to health. Although studies on structural racism have increased over the years, the selection of appropriate designs, measures, and indexes of measurement that respond to SODH has not been comprehensively documented. Therefore, the lack of evidence seems to exist. This scoping review was conducted to map and summarize global evidence on the use of various designs, measures, and indexes of measurement when studying structural racism as a social determinant of health.

**Methods:**

We performed a scoping review of global evidence from 2000 to 2022 published in 5 databases: PubMed, Cumulative Index to Nursing and Allied Health Literature (CINAHL), PsycInfo, Web of Science, ProQuest, and relevant grey literature on structural racism. We conducted a systematic search using keywords and subject headings around 3 concepts. We included peer reviewed original research/review articles which conceived the framework of social determinants of health (SODH) and studied structural racism.

**Results:**

Our review identified 1793 bibliographic citations for screening and 54 articles for final review. Articles reported 19 types of study design, 87 measures of exposure and 58 measures of health outcomes related to structural racism. 73 indexes or scales of measurement were used to assess health impacts of structural racism. Majority of articles were primary research (*n* = 43/54 articles; 79.6%), used quantitative research method (*n* = 32/54 articles; 59.3%) and predominantly conducted in the United States (*n* = 46/54 articles; 85.2.6%). Cross-sectional study design was the most used design (*n* = 17/54 articles; 31.5%) followed by systematic review (*n* = 7/54 articles; 13.0%) and narrative review (*n* = 6/54 articles; 11.1%). Housing and residential segregation was the largest cluster of exposure with the highest impact in infant health outcome.

**Conclusions:**

Our review found several key gaps and research priorities on structural racism such as lack of longitudinal studies and availability of structural or ecological data, lack of consensus on the use of consolidated appropriate measures, indexes of measurement and appropriate study designs that can capture complex interactions of exposure and outcomes related to structural racism holistically.

**Supplementary Information:**

The online version contains supplementary material available at 10.1186/s12939-022-01796-0.

## Background

Structural racism has been well documented as an important social determinant of health (SODH), the non-medical factor that influences health outcomes [1] and a key driver of health inequities [1–3] and a fundamental cause of disease [4] worldwide. Globally, structural racism is considered as a critical determinant of racial inequality in health [5–7] even more than 50 years after the ratification of the International Convention on the Elimination of All Forms of Racial Discrimination (ICERD) [8].

Although considered as an important SODH, the term ‘structural racism’ is often conflated and interchangeably used to refer institutional racism or systematic racism in public health literature. But more recently scholars have clarified this confusion asserting that they are different [9–12].

After analyzing the evolution of different terms and definitions, Bailey et al.’s definition [9] has been considered as the most contemporary definition [13]. We adopt Bailey et al.’s definition which defines.

structural racism as the totality of ways in which multiple macro structural systems (in housing, education, employment, earnings, benefits, credit, media, health care, criminal justice, and so on) and interconnected institutions mutually interact to assert biased and discriminatory policies, practices, beliefs, and distribution of resources for people in a racialized group [9, 11]. Dean and colleagues have argued that such a definition of structural racism establishes a clear distinction from: institutional racism, which associates racism within a particular type of institution, organization or in policy for/against a racial group; systemic racism, that refers to racialized systems of power; racial discrimination, which deals with practices originating from racist beliefs; and cultural racism, which upholds or reflects the values, ideologies, societal norms and practices of a particular racial group [13]. According to Jones, a) internalized racism, which refers to the internalization of oppression by a particular racial group; b) interpersonal bias or racism which refers to ‘personally mediated’ biases or racism, and c) institutional racism are 3 levels of racism [14] which can be operationalized under the larger construct of structural racism. Such mutually reinforced interactions of macro systems and institutions result in adverse health outcomes or racial inequities in health [9] which are determined by social gradient or systematic differences in health for different groups [15].

For example, according to the World Bank data life expectancy at birth in Sierra Leone [9] is 55 years whereas life expectancy at birth in Japan [10] is 84.3 years. There are less resources in Sierra Leone than in Japan leading to speculations about inequity and inaccessibility between countries.

Substantial evidence on vast racial inequities in health in the United States have been well documented [11], although racial health inequities have been a part of government statistics since the founding of colonial America [8, 12]. Any historical account of structural racism goes back to the genocide, enslavement of black people and the indigenous people by the early colonizers [9] followed by the creation of systems of racial oppression by legal initiatives such as the Jim Crow laws [11, 16].

Racial inequalities are not only in health centered organizations, but they also bleed into other organizations such as law enforcement which negatively impacts the safety and wellbeing of people of particular group.

Bor et al. for example, have documented Black Americans are ‘as nearly three times more likely than White Americans’ to be killed by police and ‘five times more likely than White Americans’ to be murdered while unarmed [17]. They are comprised of more than 40% of victims of all police homicides nationwide [17].

There has been a significant increase in the number of studies to examine the health consequences and impact pathways of structural racism in high-income countries. Dennis et al. identified eight mutually reinforcing domains of structural racism in the United States: 1) civil and political rights (including voting rights and citizenship); 2) land/housing (including neighborhoods); 3) education; 4) jobs/benefits/wealth; 5) justice system; 6) health (including health care); 7) migration and movement (including immigration, forced removal, and limited mobility); and 8) racial climate [18]. In terms of pathways between racism and health, Bailey et al. identified 9 empirical pathways in which structural racism determines health outcomes [9].

Similarly, Agénor et al. identified 843 US state laws explicitly or implicitly related to structural racism and found the 10 contemporary mutually reinforcing legal domains (i.e., voting rights laws, stand-your-ground laws, racial profiling laws, mandatory minimum prison sentencing laws, immigrant protections, fair-housing laws, minimum wage laws, predatory lending laws, laws concerning punishment in schools, and stop-and-identify laws) that directly and indirectly impact health in all 50 states and the District of Columbia from 2010 through 2013 [19].

There are numerous empirical studies that document wide range of health impacts for many historically marginalized racial/ethnic groups in the United States (e.g., African American/Black, Latinx, Hispanic, American Indian, Alaska Native, Native Hawaiian or other Pacific Islanders, Asian Americans) and other high income countries [8, 20–22].

For example, drawing on ecological studies [16–18, 23], Lane et al. identified ecological factors and potential mechanisms that led to disproportionately higher rates of heterosexually transmitted human immunodeficiency virus (HIV) among women of color in Syracuse, New York [24]. They found in Onondaga county, the tenth most populated county in New York State, that the number of African American women and Latinx women diagnosed with acquired immunodeficiency syndrome (AIDS) were nearly at 12.5 times and 9 times higher, respectively, than that of white women [24]. This study also explained how ecological factors (e.g., disproportionate and/or excessive incarceration) can lead to critical changes in population demographics (e.g., low male-to-female sex ratio in Syracuse) resulting in a ‘long term double punch’ effect (imbalanced sex ratio is likely to be associated with females choosing multiple sexual partners) in the increase of HIV transmission among African Americans [24].

Similarly, in Australia the impacts of racism, dispossession, and colonization of aboriginal Australians [25, 26] and statistically significant evidence of racist beliefs, emotions, or practices among healthcare providers in relation to minority groups [27] have been reported. Similar practices have been reported in other high-income countries such as New Zealand [28], Canada [29], Norway [30], France [31].

There are studies that document health impacts of structural racism of all sorts. Groos et al. for example, relates the health outcomes of structural racism to ‘from womb to tomb’ in their systematic review [32]. They found both mental and physical impacts including stress, anxiety, poor psychological well-being, colorectal cancer survival, myocardial infarction, mean arterial blood pressure, episodic memory function, behavioral changes, poor adherence to hypertensive treatment, and delayed HIV testing across the population [32].

It has been strongly argued in previous literature that empirical studies on structural racism and health require scientific theory, hypotheses, data, and research methods [22] in order to systematically capture the broad historical and contemporary impacts of structural racism on health [14].

Critical analysis of evidence on structural racism therefore, indicates an ever increasing scholarship on development of different theoretical frameworks (e.g. ecological, public health critical praxis, critical race theory), approaches, (e.g. sequential approach, mixed method) designs (e.g. more focus on cross-sectional design), measures (largely on common domains such as residential segregation), and indexes or scales of measurement (linear and single dimensional) to explain the etymology, pathways, health impacts and potential solutions to structural racism.

Drawing upon three frameworks: eco-social theory [33], fundamental cause theory [4, 34] and public health critical race praxis [35], Alson et al. critically examined the studies from 2000–2019 to identify studies that used quantitative measures of exposure to systemic racism in population reproductive health studies [36]. Similarly, a wide range of study designs were used in examining the health outcomes of structural racism in the context of the United States. Bor et al. for example, employed quasi-experimental study design to quantify the population-level health impacts of police killings as one of ecological factors of social determinants of health using US behavioral risk factor surveillance system (BRFSS), a nationally representative, telephone-based survey data of non-institutionalized adults aged 18 years and older [17].

Although various frameworks, study designs, measures of exposure, measures of outcome, and indexes have been used by different scholars in studying structural racism, the selection of design, measures and index of measures that responds to the framework of social determinants of health remains unclear and has not been comprehensively studied.

Several challenges have been documented in the current literature. One of the most documented challenges in undertaking empirical studies on structural racism is to develop rigorous methods to study the health impact of structural determinants of racial inequality including laws; institutional policies and practices; national, regional, state, and local economic and political infrastructures; and neighborhood and workplace conditions [24, 25, 37, 38]. For example, the use of cross-sectional design over long-term longitudinal study design on the mental health impact of structural racism has been associated with larger effect size [1]. Paradies et al. found although still statistically significant, the association between racism and health in a long-term longitudinal study with more than one year between exposure and outcome was weaker than cross-sectional or longitudinal study with relatively shorter duration [1]. Some other key methodological challenges include limitations with cross-sectional study design to make conclusion about the temporality and causality in the association between the exposure and outcome [39] and variation of estimates by the geographic unit of analysis [9, 40, 41].

The other key challenges that have been widely documented by the researchers studying structural racism include a large array of measures of exposures and outcomes [26] and measurement scales or indexes [42]. One of the most contested discussions regarding studying structural racism is to deal with the heterogeneity of definitions of measures and indexes of measurement [32]. While some scholars have preferred domain specific measures [43], other scholars have advocated for the use of index measures to study multidimensional impacts of structural racism [44]. While the most common single measure domain is residential segregation [32], the recent development is the introduction of a multidimensional measure of structural racism in Public Use Microdata Areas (PUMA) [45]. Such a heterogeneity of index of measurement indicates the lack of consensus of what index of measurement best fit in investigation of structural racism from SODH context, which calls for the need of comprehensive synthesis of indexes of measurement used in public health research.

Although there have been several studies on the health implications of structural racism, the lack of study that comprehensively maps study methods including study design, measures, and indexes in the context of SODH framework seems to exist in the literature.

Therefore, this review aimed to systematically map and summarize the global evidence on the use of different research approaches and methods and identify additional research on measures that are needed in studying structural racism as social determinant of health.

This review contributes to knowledge by providing researchers and organizations interested in combatting structural racism a comprehensive synthesis of methods, measures of exposure and health outcome and the scales that have been used in studying structural racism. This review is needed to improve the current state of research surrounding structural racism within the public health domain. This paper will guide other researchers interested in racial equity in choosing appropriate and contextual study design, measures, and scales of measurement for a research design.

## Methods

This scoping review aimed (1) to describe the literature on structural racism as it has used different study designs in studying structural racism; (2) to identify the measures of exposures and outcomes of structural racism for SODH framework; (3) to identify different measurement scales or indexes used by different by scholars, and (4) to describe different methodological challenges in studying structural racism from the framework of SODH. In undertaking this scoping review, we followed the framework of Levac et al. [46] which was based on the framework given by Arksey and O’Malley [28]. As consultation with key stakeholders, the 6^th^ step in the methodology, is optional, consultation with key stakeholders was not conducted under this current review.

### Step 1—Identify the research question

The broader research question for this review was:

What public health research methods have been used in studying structural racism as social determinant of health?

The specific research questions developed for this review were:

1. What study designs have been used in studying structural racism as social determinants of health?
2. What measures of exposure and outcome have been reported in structural racism studies?
3. What measurement scales or indexes have been used to explain the health impacts of structural racism?
4. What methodological challenges in studying structural racism as social determinants of health have been reported in the studies?

### Step 2—Identify the relevant literature

The identification of relevant literature involved a systematic search in five primary databases: PubMed, Cumulative Index to Nursing and Allied Health Literature (CINAHL), PsycInfo, Web of Science, and ProQuest. In addition, google search was used for grey literature. The development of systematic search strategy included the breaking down the broader research question into key concepts and finding key search terms for the respective concepts. Each domain of SODH and related terms were used as key word in the search strategy. To develop a comprehensive list of key words, relevant studies were reviewed. Key words were used in database to develop subject headings. A final search string including key words and subject heading terms for each concept was used. Finally, a combined search syntax with search strings of all the concepts was run in the respective database. Modifications to relevant field tagging were also done. Several rounds of consultations with the librarian throughout the development and database search took place. The systematic search across databases was conducted on 30^th^ January 2022 and was repeated on 30^th^ April 2022. The peer-reviewed English articles were considered for the review. Table 1 provides the complete search syntax for PubMed. Syntax for other databases can be available on request.

**Table 1:** PubMed Search Syntax

**Concept 1: Public Health Research Method** **Keywords:** research method, research approach, study method, study approach, public health study method, public health research method, study design, study measures, racial health disparities research, race research, critical race research, anti-racism research, anti-violence research, study tools **MeSH: “**Research Design” [MeSH], "Epidemiologic Research Design"[Mesh] **Final Search Syntax: “**Research Design” [MeSH] OR "Epidemiologic Research Design"[Mesh] OR **“**research method*” [tiab] OR “research approach*” [tiab] OR “study method*” [tiab] OR “study approach*” [tiab] OR “study design” [tiab] OR “study measure*” OR “study tools” [tiab]
**Concept 2: Structural Racism** **Keywords:** racism, systematic racism, systemic racism, structural discrimination, systematic discrimination, systemic discrimination, new racism, racial structure, racial discourse, racial practices, reverse racism, colorblind racism, institutionalized racism, internalized racism, implicit bias, structural violence, black-white disparities, racism, racial trauma, historical trauma, racial stress, racial marginalization, systemic racial disparities, structural racial disparities, institutional racial disparities, residential segregation, racial segregation, redlining, system-level discrimination, system-level racism, system-level disparities, racial bias, racial justice system, **MeSH:** "Racism"[Mesh], "Systemic Racism"[Mesh], "Race Factors"[Mesh], "Social Segregation"[Mesh], "Ethnic and Racial Minorities"[Mesh], "Bias, Implicit"[Mesh] **Final Search Syntax:** "Racism"[Mesh] OR "Systemic Racism"[Mesh] OR "Race Factors"[Mesh] OR "Social Segregation"[Mesh] OR "Ethnic and Racial Minorities"[Mesh] OR "Bias, Implicit"[Mesh] OR racism [tiab] OR “structural discrimination” [tiab] OR “systematic discrimination” [tiab] OR “systemic discrimination” [tiab] OR “new racism” [tiab] OR “racial structure” [tiab] OR “racial discourse” [tiab] OR “reverse racism” [tiab] OR “institutionalized racism” [tiab] OR “internalized racism” [tiab] OR “implicit bias” [tiab] OR “structural violence” [tiab] OR “black-white disparities” [tiab] OR racism [tiab] OR “racial trauma” [tiab] OR “historical trauma” [tiab] OR “racial stress” [tiab] OR “residential segregation” [tiab] OR “racial segregation” [tiab] OR redlining [tiab] OR “racial bias” [tiab]
**Concept 3: Social determinants of Health** **Keywords:** Determinants of health, environmental factors of health, social factors of health, built environment for health, behavioral determinants of health, social conditions for health, social services, social spending, social welfare, housing, education, income support, nutrition, food stamp, SNAP, public safety, transportation, health outcomes, health savings, health costs, health spending, health expenditure, structural determinants of health, determinants of mental health, social determinants of mental health, **MeSH:** "Social Determinants of Health"[Mesh], "Built Environment"[Mesh], "Social Welfare"[Mesh], "Housing Instability"[Mesh], "Food Assistance"[Mesh], "Health Expenditures"[Mesh] **Final Search Syntax:** "Social Determinants of Health"[Mesh] OR "Built Environment"[Mesh] OR "Social Welfare"[Mesh] OR "Housing Instability"[Mesh] OR "Food Assistance"[Mesh] OR "Health Expenditures"[Mesh] OR “determinants of health” [tiab] OR “social service*” [tiab] OR “social spending” [tiab] OR “social welfare” [tiab] OR housing [tiab] OR education [tiab] OR “income support” [tiab] OR nutrition [tiab] OR “food stamp” [tiab] OR SNAP [tiab] OR “public safety” [tiab] OR transportation [tiab] OR “health outcomes” [tiab] OR “health savings” [tiab] OR “health costs” [tiab] OR “health spending” [tiab] OR “health expenditure” [tiab] OR “structural determinants of health” [tiab] OR “determinants of mental health” [tiab] OR “social determinants of mental health” [tiab]

### Step 3—Select the literature

Bibliographic citations which were retrieved through the electronic databases were imported into Rayyan [29], a systematic review management software. Bibliographic databases were reviewed and duplicates were removed. Two stage relevance screening was conducted using the inclusion criteria (Table 2).

**Table 2:** Inclusion and exclusion criteria used to identify the articles for the review

Inclusion criteria	Exclusion criteria
▪ Article conceived the framework of social determinants of health (SODH)	▪ Conference abstracts or proceedings
▪ Structural racism studied as SODH	▪ Review protocols
▪ Studies on any health outcomes related to structural racism	▪ Commentary articles
▪ Original research of any study design	▪ Recommendation articles
▪ Systematic review	▪ Articles in media
▪ Peer reviewed articles	▪ Calls for research
▪ PhD thesis	▪ Literature reviews without a search strategy
▪ Published from 2000-2022 (till the search date)	▪ Letters to the editor
▪ Studies conducted in high-income countries	▪ Book reviews, textbooks, replies from author, erratum, or opinion pieces
	▪ Published prior to the considered deadline
	▪ Studies that did not report on health outcomes

At first, titles and abstracts were screened. Secondly, full texts of potentially relevant articles were screened. Articles were independently screened by two reviewers (MKA and DS) at both stages of screening. Articles were relevant to this review if they considered social determinants of health (SODH) framework or any other theoretical framework relevant to SODH and studied structural racism as a determinant of health.

The articles that were considered ineligible by one reviewer were cross examined by the 2^nd^ reviewer. At the second level the reviewers discussed the papers one by one for the ones they disagreed with for exclusion. Article was included when two reviewers reached to an agreement.

### Step 4—Chart the data

A priori structured data charting matrix was developed by MKA, reviewed by DS and supervised by SDL. The development of data charting matrix was done in accordance with broader and specific research questions. The charting matrix captured information from studies on year of publication, country of study, study objective, theoretical concepts or frameworks, study design, measures of exposure and outcome, measurement scale or indexes, data analysis plan, key findings and methodological challenges reported in the study. After charting data from all studies, the second reviewer screened the matrix for data accuracy.

### Step 5 – Collate, summarize, and report results

All 54 articles were charted by the year of publication, study objective, theoretical concepts or frameworks, study design, measures of exposure and outcome, measurement scale or indexes, data analysis plan, key findings and methodological challenges reported in the study. For the geographical location the study setting was considered. For review article the country of the first author was considered. Descriptive statistics using frequency counts and percentages was used to depict an overview of the characteristics of the studies. For the measures of exposure and outcome, cluster plotting with the help of Microsoft Excel was done to illustrate the dominant health implications of structural racism. Key findings related to the specific research questions were summarized narratively.

## Results

### Study characteristics

The search strategy yielded 1,793 articles. After the removal of the duplicates, 1,542 articles remained for the title and abstract screening. Reviewers screened the titles and abstracts of 1,542 articles and dropped 1,480 articles. 62 articles were taken for full text review. Individual hand search of all studies which led to an additional 4 articles was also done. During the full text review 12 articles were excluded. This left 54 articles for final review.

**Fig. 1:**
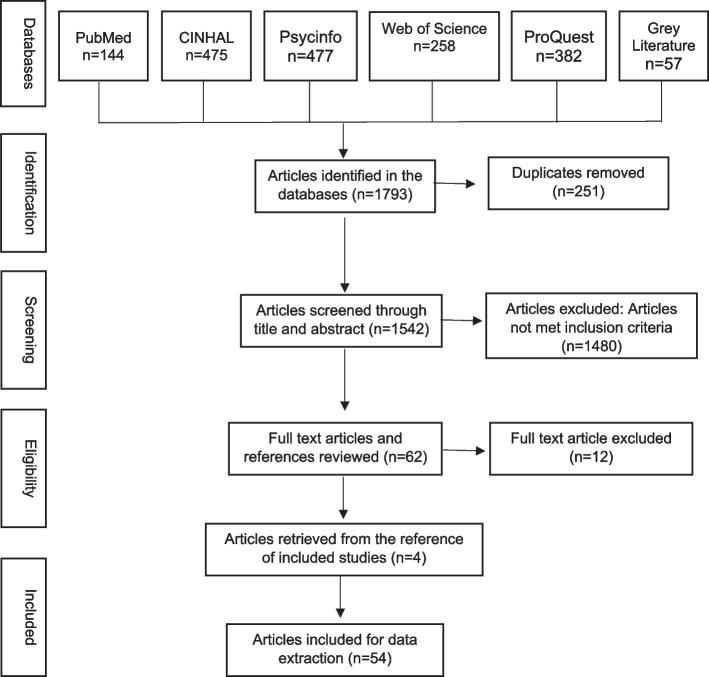
Flow diagram of the article screening process

**Fig. 2:**
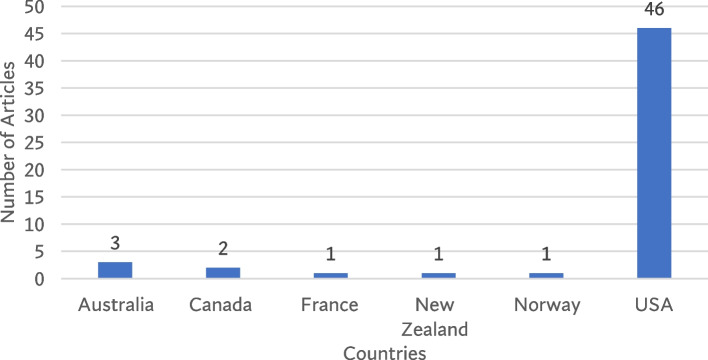
Countries of publication of included studies (*n* = 54) from 2000–2022

Figure 1 shows the flow diagram of the process of inclusion and exclusion of the studies for this review. Articles included in this review reported or discussed a wide range of public health exposures and outcomes of structural racism, which was captured employing different study designs, theoretical frameworks, scales of measurements. The review found that majority of the included articles were primary research (*n* = 43/54 articles; 79.6%), conducted in the United States (*n* = 46/54 articles; 85.2.6%), and predominantly used quantitative research method (*n* = 32/54 articles; 59.3%, Fig. 2).

**Fig. 3:**
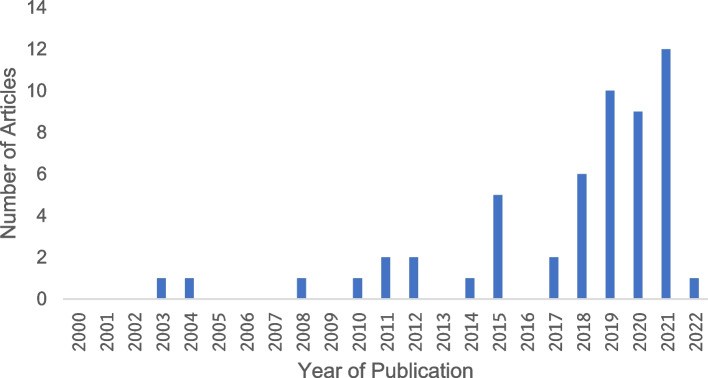
Year of publication of included studies (*n* = 54) from 2000–2022

The majority of the studies used theoretical frameworks which conceived the SODH approach (*n* = 36/54 articles; 66.7%; Table 3). The review also found that the number of studies has increased substantially since 2015 (Fig. 3).

**Table 3:** Characteristics of included studies (*n* = 54)

Characteristics	N (%)
**Year of Publication**	
2000–2005	2 (3.7)
2006–2010	2 (3.7)
2011–2015	10 (18.5)
2016–2021	40 (74.1)
**Countries of Publication**	
Australia	3 (5.6)
Canada	2 (3.7)
France	1 (1.9)
New Zealand	1 (1.9)
Norway	1 (1.9)
USA	46 (85.2)
**Study Type**	
Primary research	43 (79.6)
Review	9 (16.7)
Doctoral thesis	2 (3.7)
**Study Approach**	
Community collaborative participatory research	3 (5.6)
Mixed Method	3 (5.6)
Qualitative	4 (7.4)
Quantitative	32 (59.3)
Review	12 (22.2)
**Study Design**	
Quantitative	
Case	1 (1.9)
Case–control	1 (1.9)
Cohort	2 (3.7)
Cross-sectional	17 (31.5)
Ecological	2 (3.7)
Longitudinal cohort	2 (3.7)
Longitudinal randomized controlled trial	1 (1.9)
Non-experimental survey	3 (5.6)
Quasi-experimental	1 (1.9)
Retrospective cohort	1 (1.9)
Sequential quantitative and qualitative	1 (1.9)
Qualitative	
Exploratory	3 (5.6)
Grounded theory	1 (1.9)
Review	
Integrative Review	1 (1.9)
Narrative Review	6 (11.1)
Systematic Review	7 (13.0)
Systematic Review & Meta Analysis	2 (3.7)
Others	
Policy Surveillance	1 (1.9)
**Study Population**	
By ethnicity & gender	
Black women	7 (13.0)
Black & Hispanic women	1 (1.9)
Black & Latin women	1 (1.9)
Black & White women	2 (3.7)
Black, Hispanic & White women	1 (1.9)
Black, Asian, Native American women & LGBTQ adults	1 (1.9)
By ethnicity & general population	
Aboriginal people	6 (11.1)
Black adults	4 (7.4)
Black & White adults	4 (7.4)
Black, Hispanic & White adults	1 (1.9)
Black, Latin & White adults	2 (3.7)
Black, Asian, Hispanic, Latino, Native American & White adults	3 (5.6)
People of color adults	7 (13.0)
By category	
Black K-12 grade students	3 (5.6)
Healthcare providers	5 (9.3)
NGO professionals	2 (3.7)
People with disability	1 (1.9)
Other	3 (5.6)
**Measures of Exposure**	
Studies reported at least 1 exposure	21 (38.9)
Studies reported 2–4 exposures	18 (33.3)
Studies reported 5–7 exposures	10 (18.5)
Studies reported 8–9 exposures	3 (5.6)
Studies reported 10–12 exposures	2 (3.7)
**Measures of Outcome**	
Studies reported at least 1 outcome	49 (90.7)
Studies reported 2–3 outcome	5 (9.2)
Studies reported 4–5 outcome	2 (3.7)
**Use of measurement index**	
Studies used no index	17 (31.5)
Studies used index	37 (68.5)

In terms of study designs the most preferred study design in studying structural racism was cross-sectional (*n* = 14/54 articles; 31.5%). Black women by ethnicity and gender (*n* = 7/54 articles; 13.0%) and people of color adults in general (*n* = 7/54 articles; 13.0%) were the most studied study population. It was also found that majority of the studies (*n* = 39/54 articles; 72.2%) reported at least 1–4 measures of exposure of structural racism and almost all the studies (*n* = 49/54 articles; 90.7%) reported at least 1 outcome measure of structural racism as social determinant of health. Similarly, majority of the studies (*n* = 37/54 articles; 68.5%) used measurement index in capturing the association between structural racism and health.

### What study designs have been used in studying structural racism as social determinants of health?

The review found 19 types of study design that have been used in studying structural racism as social determinant of health in the developed country context. It is clearly noticeable that the cross-sectional study design was the most commonly used design (*n* = 17/54 articles; 31.5%) [22, 26–32, 39, 47–54] followed by systematic review (*n* = 7/54 articles; 13.0%) [23, 40, 41, 43, 44, 55] and narrative review (*n* = 6/54 articles; 11.1%) [18, 45, 46, 56–58] (Fig. 3). The cross-sectional study design was used to examine a wide range of exposure clusters of structural racism including access to healthcare [22, 26, 53], civil and legal system discrimination [29, 53], educational attainment [26–29, 31, 32, 51, 53], employment and income [30, 39, 41, 43, 47, 48, 50, 53, 54], health coverage [51], health status [26, 49], housing and residential segregation [22, 26, 30, 31, 51, 53], incarceration [22, 29], structural violence [22], sociodemographic characteristics [26, 28, 39, 47, 50, 52], socioeconomic status [26, 29, 31], institutional and personal discrimination [54], racial discrimination [48, 50] and immigration related discrimination [28]. Similarly, the clusters of health outcome measures that were examined by cross-sectional design included infant health outcomes [26, 29, 30, 50], chronic conditions [27, 47, 48, 53], access and quality of healthcare [49, 52], quality of life [30, 47, 48], communicable diseases [22, 32], and mental health [28, 31].

**Fig. 4:**
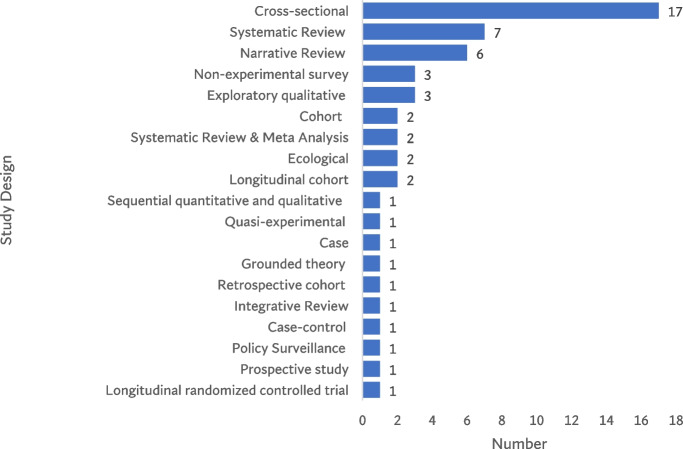
Distribution of study designs of included studies (*n* = 54) from 2000–2022

The other study designs that were found in the studies include case (*n* = 1/54 articles; 1.9%) [59], cohort (*n* = 2/54 articles; 3.7%) [60, 61], ecological (*n* = 2/54 articles; 3.7%) [62, 63], longitudinal cohort (*n* = 2/54 articles; 3.7%) [64, 65], longitudinal randomized controlled trial (*n* = 1/54 articles; 1.9%) [66], non-experimental survey (*n* = 3/54 articles; 5.7%) [67–69], prospective study (*n* = 1/54 articles; 1.9%) [70], quasi-experimental (*n* = 1/54 articles; 1.9%) [11], retrospective cohort (*n* = 1/54 articles; 1.9%) [71], sequential quantitative and qualitative (*n* = 1/54 articles; 1.9%) [72], exploratory qualitative (*n* = 3/54 articles; 5.7%) [73–75], grounded theory (*n* = 1/54 articles; 1.9%) [76], integrative review (*n* = 1/54 articles; 1.9%) [77], meta-analysis (*n* = 2/54 articles; 3.7%) [78, 79], and policy surveillance (*n* = 1/54 articles; 1.9%) [13] (Fig. 4).

### What measures of exposure and outcome have been reported in structural racism studies?

**Fig. 5:**
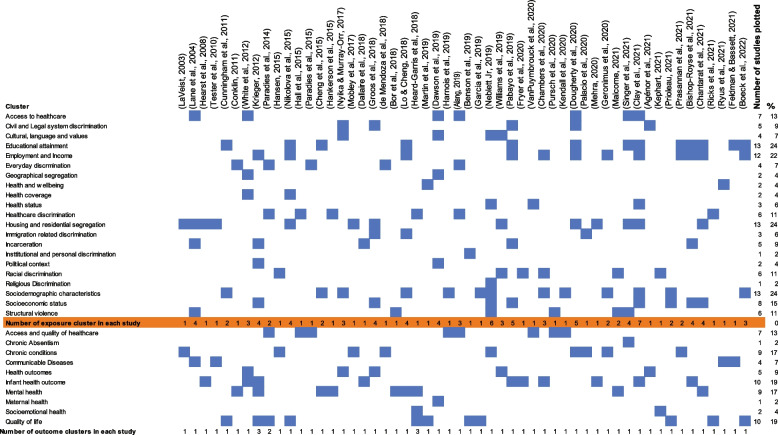
Cluster plotting of measures of exposure and measures of outcome in the included study (*n* = 54) from 2000–2022

It was found that a total of 87 measures of exposure have been reported in all 54 studies. The following paragraph addresses racial bias and/or discrimination as they relate to a wide number of variables. Please see the additional data file for a full list of exposure and outcome (Fig. 5).

### Exposure

This list of measures of exposure was  grouped in 20 clusters: access to healthcare (*n* = 7), civil and legal system discrimination (*n* = 5), cultural, language and values (*n* = 4), educational attainment (*n* = 13), employment and Income (*n* = 12), everyday discrimination (4), geographical segregation (*n* = 2), health and wellbeing (*n* = 2), health coverage (*n* = 2), health status (*n* = 3), healthcare discrimination (*n* = 6), housing and residential segregation (n = 13), immigration related discrimination (*n* = 3), incarceration (*n* = 5), Institutional and personal discrimination (*n* = 1), political context (*n* = 2), racial discrimination (*n* = 6), religious discrimination (*n* = 1), sociodemographic characteristics (*n* = 13), socioeconomic status (*n* = 8) and structural violence (*n* = 6).

Access to healthcare cluster includes specific measure of exposure such as asthma rate [62], access to healthcare during pregnancy [26], maternity care system [77], health facility based segregation [46], health care [53], accessibility barriers [72], constraints on access to sexually transmitted diseases (STD) services [22].

The list of reported exposures related to civil and legal system discrimination include legal regulation [54), voting rights [13], stand your-ground laws [13], racial profiling laws [13], mandatory minimum prison sentencing laws [13], immigrant protections [13], fair-housing laws [13], minimum-wage laws [13], predatory lending laws [13], laws concerning punishment in schools [13], stop-and identify laws [13], criminal justice [23, 53], home mortgage discrimination [23], juvenile custody rate [29], sentencing rates [29] and capital punishment [29].

The educational attainment cluster remains to be one of the largest clusters reported by the highest number of studies (*n* = 13). Reported as one of the most important social determinants of health, educational attainment cluster includes individual education level and attainment [26, 28, 29, 51, 53, 63, 68, 70], high school education, math and English test score [62], parental education [80], education inequality [31] and school stability rate [62].

Similarly, employment-income cluster was reported as another important  determinant of health for the people of color in general (*n* = 12) through the exposure of having/not having employment [18, 26, 27, 29, 51, 53], employment inequity [31], kind/status of employment [68] household income [27, 29, 68], individual income [26, 50, 63], income inequity [31], income to needs ratio [3], poverty [18, 47, 68].

Housing and residential segregation cluster was also reported by highest number of studies through number of exposures of structural racism including government help for rent [26], household characteristics and conditions [51, 53, 59], length of residence in the neighborhood [26], neighborhood safety [26], residence in public housing [26], residential housing pattern [23], racial housing segregation [22, 30, 31, 57, 61, 69, 79], residential vacancy rate [62].

Sociodemographic characteristics including age [39, 56, 68, 70], gender [28, 45, 67, 70], marital status [26, 28], race/ethnicity [39, 45, 50, 52, 65, 68, 70, 76], race related stress [67] have also been widely reported cluster of exposure in all 54 studies. The other widely reported exposure cluster was socioeconomic status which included car ownership (65), class consciousness (77), homeownership [26, 31], occupational status [29, 63], and wealth [18, 23, 67].

### Outcome

In terms of outcome 58 measures of health outcomes in which exposures to racism had measurable impacts on health were reported in the included studies. These 58 measures were categorized into 10 clusters of outcome measures: access and quality of healthcare (*n* = 7) [40, 49, 52, 55, 72, 76], chronic absenteeism [62], chronic conditions (*n* = 9) [27, 47, 48, 53, 56, 61, 64, 69, 71], communicable diseases (*n* = 4) [19, 55, 63, 79], general health outcomes (*n* = 5) [15, 42, 58, 59, 61], infant health outcome (*n* = 10) [18, 26, 29, 30, 43, 50, 60, 63, 79, 81], mental health (*n* = 9) [11, 18, 28, 31, 43, 58, 65, 66, 78], maternal health (*n* = 1) [77], socioemotional health (*n* = 1) (74), quality of life (*n* = 10) [1, 39, 41, 44, 51, 54, 67, 68, 70, 73].

Among all the clusters infant health outcome and quality of life were mostly affected by structural racism. The infant health outcome included preterm birth [43, 50, 60], low birth weight [26], preterm birth and low birth weight [50, 79], neonatal outcome and home environments [81], infant mortality [18, 30, 43], and cortisol reactivity [43]. The quality of life was reported through the measures of health outcomes including dementia [39], disability pattern [51], everyday experiences of discrimination [70], green space in the neighborhood [41], health and wellbeing [54, 73], increased self-awareness [44], mental health [39, 40], wellness score [67], years of life loss [68].

Chronic conditions such as Framingham risk score (FRS) for cardiovascular disease [71], acute respiratory syndrome [27], allostatic load [47], body mass index [53], DNAm (methylation) [64], late-stage diagnosis of cancer [69], number of chronic conditions [61], psychological (e.g., anger, fear) stress responses [56] and waist circumference [48] were reported to be associated with structural racism.

### What measurement scales or indexes have been used to explain the health impacts of structural racism?

The review found that majority of the studies used measurement scales or indexes of structural racism (*n* = 37/54 articles, 68.5%) while 17 (31.5%) studies used no scale of measurement. The total number of indexes that were reported in 37 studies was 73. Concentration of Extremes [29, 51, 63] Dissimilarity Index [23, 31, 41], Everyday Discrimination Scale (EDS) [1, 18, 43, 52], Experience of Discrimination Scale (EDS) [1, 18, 43, 50, 70], Five Segregation Scale [46, 79], Index of Race Related Stress (IRRS) [23, 56, 67, 78], Isolation Index [23, 30, 69] and Perceived Racism Scale (PRS) [1, 78] were the most used scales of measurement in the studies (Table 4).

**Table 4:** Full list of Indexes reported in the studies

Measurement Scale/Indexes	References
1. Affective Racial Attitude Scale	[44]
2. Akaika Information Criteria (AICc)	[48]
3. Anger and Hopelessness Scale	[43]
4. Beck Depression Inventory Scale	[70]
5. Census Track based Reddling Index	[42]
6. Census Track Neighborhood Crosswalk	[72]
7. Census track Socioeconomic Disadvantage Index	[42]
8. Cognitive Function Score Index	[52]
9. Community Belonging Scale	[70]
10. Community Violence Scale	[70]
11. Concentration of Extremes Index	[29, 51, 63]
12. Contemporary Racism Awareness Scale	[44]
13. County Structural Racism Index	[50]
14. Deferred Action for Childhood Arrivals (DACA) status	[42]
15. Dichotomized Scale	[31]
16. Dissimilarity Index	[23, 31, 41]
17. Epidemiological Studies Depression Scale	[69]
18. Ethnic Attitude Scale	[44]
19. Everyday Discrimination Scale (EDS)	[1, 18, 43, 52],
20. Everyday Unfair Treatment Scale	[43]
21. Experiences of Discrimination (EOD) Scale	[1, 18, 43, 50, 70]
22. Feelings of Warmth Scale	[44]
23. Felony Incarcerations	[42]
24. Five Segregation Scale	[46, 79]
25. Framingham risk score (FRS)	[75]
26. Frequency of Discrimination Experience Index	[54]
27. Gendered Racial Microaggression Scale	[71]
28. Generic Survey Index	[42]
29. Geographical Location Index	[13]
30. Global Moran Index (I)	[48]
31. Hardship Index	[67]
32. Implicit Association Test-IA	[44]
33. Index of macro-level factors	[66]
34. Index of Race Related Stress (IRRS)	[23, 56, 67, 78]
35. Index of race related stress-brief and five factor wellness Inventory	[42]
36. Institutional Racism subscale of the Index of Race-Related Stress-Brief Version (IRRS-B)	[42]
37. Isolation Index	[23, 30, 69]
38. Knowledge and Attitude towards Immigrants Scale	[44]
39. Legal Coding Scheme Index	[5]
40. Likert Scale	[32]
41. Local Moran's Index (LISA)	[48]
42. Major Life Discrimination [MLD] Scale	[68]
43. Measurement Instrument	[56]
44. Multicultural Counseling Knowledge and Awareness Scale	[44]
45. Multidimensional inventory of black identity (MIBI): public regard subscale	[1]
46. Nadanolitization scale	[1]
47. Negative Social Interaction Scale	[43]
48. Neighborhood Satisfaction Scale	[43]
49. New Racism Scale	[44]
50. Perceived Ethnic Discrimination Questionnaire (PEDQ)	[1]
51. Perceived Lifetime Discrimination Scale	[69]
52. Perceived Racism Scale (PRS)	[1, 78]
53. Residential Redlining Index	[42]
54. Racial Discrimination Scale	[42]
55. Racial Preference Scale	[44]
56. Racism and Life Experience Scale—Brief Version (RaLES-B)	[82]
57. Racism and life experience scales (RaLES)	[1]
58. Racism Reaction Scale (RRS)	[82]
59. Redlining Index of Mortgage Discrimination	[42]
60. Relative Proportion Index	[42]
61. Scale on Beliefs about Race Related Policies	[44]
62. Scale on Race-based Meritocracy	[44]
63. Scale on Self-Perception on Racism among Providers	[44]
64. Schedule of Racist Life Events (SRE) and Perceptions on Racism Scale (PoRS)	[1, 82]
65. Segregation index	[65]
66. Self- Reported Scale	[42]
67. Semantic Differential Situational Attitude Scale	[44]
68. Social Distance Scale	[44]
69. Survey Instrument	[51]
70. Vignettes	[44]
71. Visible Ethnic Identity Attitude Scale	[44]
72. White Racial Identity Attitude Scale	[44]
73. Zone Improvement Plan (ZIP)	[44]

### What methodological challenges in studying structural racism as social determinants of health have been reported in the studies?

It was found that 44 (*n* = 44/54 articles; 81.4%), studies discussed methodological challenges related to studying structural racism as social determinant of health. The most widely reported methodological challenges were found to relate to study design (*n* = 9/54 articles; 16.7%) [11, 26, 27, 43, 48, 51, 53, 55, 62], scales of measurement (*n* = 9/54 articles;16.7%) [18, 26, 39, 52, 53, 56, 63, 70, 81], measures of exposure (*n* = 8/54 articles; 14.81%) [40, 46, 49, 53, 56, 61, 67, 79], and data analysis approach (*n* = 5/54 articles; 9.2%) [1, 30, 31, 50, 78].

The other methodological challenges were related to sample size and sampling method (*n* = 3/54 articles; 5.5%) [54, 64, 66, 68], study population (*n* = 4/54 articles; 7.4%) [40, 65, 74, 76], study duration (*n* = 1/54 articles; 1.9%) [13], study approach (*n* = 3/54 articles; 3.5%) [45, 72, 73], lack of availability of data on structural level (*n* = 2/54 articles; 3.7%) [57, 68], weighted SODH score (*n* = 1/54 articles; 1.9%) [71], use of secondary data (*n* = 1/54 articles; 1.9%) [28], bias, confounding and misclassification (*n* = 2/54 articles; 3.7%)[29, 32].

Although cross-sectional was commonly used study design, it was associated with several methodological challenges and limitations in terms of temporal ordering of variables, biases towards type II errors for physical outcomes. Therefore, it has been argued that causality cannot be understood from cross-sectional studies [48]. Hall et al. found cross-sectional study design with limited ability to explain predictive relationships for chronic conditions between a risk factor (e.g., exposure to a biased health care provider) and an outcome (e.g., a patient’s psychological distress) related to structural racism [55]. Dougherty et al. has documented similar observations on the use of cross-sectional study design [53]. Clay et al., on the other hand, found cross-sectional design particularly suitable for ‘fragile or risk population’ such as non-Hispanic White and Black unmarried women with lower educational attainment where women were found to have  low-birth weight infants. Retrospective design was also associated with limitation [27]. However, ecological design was considered suitable and effective to examine association and correlation for macro level factors [22, 62].

Several methodological challenges with different scales of measurement were reported in several studies. The most widely documented challenge was the reliability issue of the use of self-reporting data with Experiences of Discrimination (EOD) and EDS scale of measurement [18]. The other methodological issues related to the different scales of measurement include lack of sensitivity to non-uniform difference score and inflated Cronbach's Alpha for internal consistency reliability in studying internalized discrimination [70], the use of secondary data based on pre-designed questionnaire in studying incarceration [81], lack of construct validity in measuring the latent variable in the confirmatory factor modeling [53], narrow focus of the scales of measurement on the individual along with lack of validated measures of institutional, cultural and structural racism [56]. The lack of longitudinal studies to examine the multiple pathways and dimensional aspects of structural racism and its health outcomes has also been reported [56].

In terms of measures of exposure, residential segregation has been associated with difficulties and potential measurement error [79] as it has not been easy to identify the right kind of measures in examining segregation and its health outcomes. White et al. found it unclear whether to select direct measures versus proxy measures in understanding segregation and health outcomes [46]. Direct or explicit measures with one item have been associated with social desirability bias [32]. The inclusion of one category of study participant such as aboriginal women in New Zealand [81], physicians [44], non-government organization staffs [78] and one ethnic category [54] in the United States context have been associated with non-conclusive and non-generalizable findings.

Other reported methodological issues include lack of structural level data [61], bias, confounding and misclassification [39] due to unavailability of study participants’ in-depth information such as individual income information, low standardized entropy for our latent class model and lack of control potential confounders in analysis [41], unvalidated and weighted SODH score for cardiovascular events [75], and the inability of correlational data to explain causal relationship between exposure and outcome [82].

See full description of the included studies in Table 5 (additional file).

**Table 5:** Full description of the included studies

**Authors/Year**	**Objectives**	**Study Concepts for Exposure**	**Study Concepts for Outcome**	**Study Population**	**Method/Study Design **	**Measures of Exposure**	**Measures of Outcome**	**Indexes/** **Scales of Measurement **	**Findings **
Agénor et al. (2021) [19]	To develop a comprehensive, longitudinal database of state laws that are explicitly or implicitly related to structural racism for various marginalized racial/ethnic groups (e.g., Black, Indigenous, and Latinx populations)	Legal systems, structural racism	Health outcome	State laws	Quantitative/ Policy Surveillance	10 contemporary legal domains (voting rights (33), stand your-ground laws (34), racial profiling laws (35), mandatory minimum prison sentencing laws (36), immigrant protections (37), fair-housing laws (38), minimum-wage laws (39), predatory lending laws (40), laws concerning punishment in schools (41), and stop-and identify laws (42)	Health Outcome (1)	Legal Coding Scheme (39)	843 US state laws were found explicitly or implicitly related to structural racism across the 10 contemporary legal domains (ie, voting rights laws, stand-your-ground laws, racial profiling laws, mandatory minimum prison sentencing laws, immigrant protections, fair-housing laws, minimum wage laws, predatory lending laws, laws concerning punishment in schools, and stop-and-identify laws) in all 50 states and the District of Columbia from 2010 through 2013
Alang (2019) [83]	To characterize unmet need by identifying characteristics of blacks that are associated with reporting different reasons of perceived unmet need for mental health care	Racism	mental health	African American adults	Mixed method/ Sequential quantitative and qualitative	Cost (54), stigma (55), Minimization (56), low perceived effectiveness of treatment (57), accessibility barriers (12)	Unmet need of healthcare (2)	NA	Higher education was associated with greater odds of reporting stigma and minimization of symptoms as reasons for unmet need and racism causes mistrust in mental health service systems.
Benson et al. (2019) [71]	To examine reported experiences of discrimination against African American, Asian American, Native Americans, women and LGBTQ adults	Experience of discrimination	Health outcome	African American, Asian American, Native Americans, women and LGBTQ adults	Quantitative/ Cross-sectional	Institutional and interpersonal discrimination (58)	Identity based discrimination (3)	Survey Instrument (69)	In healthcare settings, 32% of African American, 23% native Americans, 20% Latinos, 13% Asian Americans, 38% native Americans reported identify based discrimination.
Bishop-Royse et al. (2021) [84]	To examine associations between infant mortality rates (IMRs) and measures of structural racism and socio-economic marginalization in Chicago, Illinois	Structural racism, economic marginalization	Infant mortality	Community residents	Quantitative/ Ecological	incarceration (61), educational attainment (11), income (15), and occupational status (81)	Infant mortality (4)	Index of Concentration at the Extremes (ICE) (11), Hardship Index (31)	Community areas with the lowest ICERace scores (those with the largest concentrations of Black residents, compared with White) had IMRs that were 3.63 times higher than those communities with the largest concentrations of White residents.
Boeck et al. (2021) [85]	To examine deaths attributable to violence and chronic diseases by area based social factors	Structural social factors	Deaths	Residents	Quantitative/ Non-experimental survey	Age (24), sex (23), race/ ethnicity (22), education level (11), employment status (14), median household income (MHI) (74), and percent below poverty level (PBPL) (60)	Years of life loss (5)	Census tract neighborhood crosswalk (6)	For chronic diseases and homicides, AYLLs increased as a neighborhood’s percent Black, below poverty level, unemployment, and below high school education increased
Bor et al. (2018) [17]	To estimate the impact of police killings of unarmed black Americans on self-reported mental health of black American adults in the US general population.	Police killing	mental health	African American	Quantitative/ Quasi-experimental	Number of police killing of unarmed black Americans in the 3 months prior BFRSS interview (63)	Number of days with 'not good' mental health status (6)	Geographical location index (29)	Each additional police killing of an unarmed black American was associated with 0·14 additional poor mental health days (95% CI 0·07–0·22; p=0·00047) among black American respondents.
Chambers et al. (2020) [67]	To describe pregnant and early post-partum Black women's exposure to structural racism and self-reported experiences of racial discrimination, and the extent to which these factors are related	Structural racism, racial discrimination	Neonatal health	Black women	Quantitative/ Cross-sectional	Race (22), Experiences of discrimination (45) and income (15)	Preterm birth (7) and Low birth weight (8)	Concentration of Extremes Index (11) & Experience of Discrimination Scale (21)	Living in highly deprived race and income neighborhoods was associated with experiencing racial discrimination in three or more situational domains. Black women are exposed to high levels of racism that may have negative impacts on maternal health outcomes.
Chantarat et al. (2021) [45]	To examine the multidimensional measures of structural racism using a latent class model	Structural racism	Covid 19 vaccination	PUMA Residents	Quantitative/ Cross sectional	Residential segregation (48), education inequity (11), Employment inequity (14), home ownership inequity (18), income inequity (15)	covid 19 vaccination rates (9)	Index of dissimilarity	Statistically significant differences due to structural racism by vaccination rates were observed between PUMAs with high and low Black-White income inequity only (7.2% vs 5.3%, p=.001)
Cheng et al. (2015) [80]	To describe levels of perceived lifetime discrimination among young adults and determine its role in understanding this racial/ethnic disparity	Perceived lifetime discrimination	Depression	African American 5-12 graders	Quantitative/ Longitudinal cohort	Race (22) and Parental Education (82)	Depression (10)	Perceived lifetime discrimination scale (51), Epidemiological Studies Depression Scale (17)	Black students from professionally educated families had the greatest discrimination scores, 1.8 times greater than among their white peers (mean Black = 42.1 vs mean White = 22.8; P < .0001); Greater parental education was associated with lower depressive symptoms in all regression models.
Clay et al. (2021) [65]	To explore racial differences in influential sociodemographic, economic, and environmental factors in women with a low-birth-weight infant	Sociodemographic, economic, and environmental factors	Low Birth Weight (LBW)	Non-Hispanic Black and White women	Quantitative/Cross-sectional	Marital Status (10), Educational level (11), access to healthcare during pregnancy (12), health status (13), employment (14), income (15), government help for rent (16), residence in public housing (17), homeownership (18), car ownership (19), neighborhood safety (20), length of residence in the neighborhood (21)	Low Birth Weight (8)	NA	For non-Hispanic Blacks, being married (OR=.55, P=0.003), having health care coverage (OR=.35, P<0.001), and living in public housing (OR=.64, P=0.031) were associated with a decreased likelihood of having LBW infants were 1.54 times (P=0.010) more likely to have LBW infants, as compared to NH Whites
Conklin (2011) [86]	To examine the association between perceived racism and mental health	Perceived racism	mental health	African American adults	Review/ Systematic Review and Meta Analysis	Perceived racism (58)	Mental health (11)		Higher instances of perceived racism were associated with lower levels of mental health
Cunningham et al. (2011) [87]	To examine the differential item functioning related to race, gender, age and educational attainment	Socio demographic factors	Experiences of Discrimination (EOD)	Young adult	Quantitative/ Prospective Study	Race (22), Gender (23), Age (24), Educational attainment (11)	Everyday Experiences of Discrimination (12)	Experiences of Discrimination Index (21)	Race and Gender were statistically significantly associated with EOD at school, getting a job, getting a house and in public place.
Dallaire et al. (2018) [88]	To examine the impact of mother or partner incarceration during pregnancy on neonatal outcomes and home environments	Parental incarceration	Adverse childhood experience	African American Mothers	Quantitative/ Case Control	Parental Incarceration (49)	Neonatal outcome (13) and home environments (25)	NA	The women who experienced incarceration of themselves or their husband/partner were significantly less likely to deliver an LBW infant and more likely to live in a home with a loaded firearm in the home.
Dawson et al. (2019) [28]	To examine the ‘causes of the causes’ of maternal inequity specific to New Zealand, and explain factors underlying continuing disparity, despite a free, women centered, continuity of care maternity system.	Social contributors	Maternal health inequity	African American Mothers	Review/ Integrative Review	Ethnicity- Race- Cultural factors (25), Geographical access (26), political context (27), maternity care system (28), acceptability (29), colonialism (30)	Maternal health (11)	NA	Six integrated factors – Physical Access, Political Context, Maternity Care System, Acceptability, Colonialism, and Cultural factors – were identified as barriers to equitable maternal health in Aotearoa New Zealand. A complex set of underlying structural and systemic factors, such as institutionalized racism, serve to act as barriers to equitable maternity outcomes and experiences.
de Mendoza et al. (2018) [89]	To examine the influence of perceived racism and discrimination on DNAm in a sample of African American mothers enrolled in the Intergenerational Impact of Genetic and Psychological Factors on Blood Pressure (InterGEN) study	Perceived racism & discrimination	DNA methylation (DNAm)	Mother/child dyads	Quantitative/ Longitudinal cohort	Major Life Discrimination [MLD] (87) & Race-Related Events [RES] (22)	DNAm (epigenome-wide association study [EWAS]) (14)	Major Life Discrimination [MLD] Scale (42) & Race-Related Events [RES] scales)	After controlling for age, smoking, and cell composition, MLD was significantly associated with DNAm at nine CpG (regions of DNA where a cytosine nucleotide is followed by a guanine nucleotide) sites (false discovery rate [FDR]-corrected p < .05). significant epigenetic associations between disease-associated genes (e.g., schizophrenia, bipolar disorder, and asthma) and perceived discrimination as measured by the MLD Scale.
Dougherty et al. (2020) [70]	To examine the association between the structural racism and BMI for black and white men and women	Structural racism, obesity	BMI	White and black adults	Quantitative/ Cross-sectional	Housing (52), education (11), employment (14), health care (12), and criminal justice (51)	Body mass index (BMI) (15)	County structural racism index (13)	County structural racism was associated with larger increases in BMI among black men than black women. County structural racism was associated with reduced BMI for white men and no change for white women
Feldman & Bassett (2021) [63]	To measure inequality in COVID-19 mortality jointly by race and ethnicity and educational attainment	Educational attainment, race/ethnicity	inequality in covid 19 mortality	Persons aged 25 years or older	Quantitative / Cross-sectional	Education (11) and Race (22)	COVID 19 mortality (16)	NA	Age-adjusted cumulative mortality rates for the overall population were highest among persons with the lowest educational attainment (208.1 per 100 000 population; Racial and ethnic minority women died at higher rates than non-Hispanic White men of the same age group, with the exception of non-HispanicAsian women. [95% CI, 207.3-208.9 per 100 000 population]).
Fryer et al. (2020) [81]	To investigate the prevalence of self-reported discrimination and its association with the prevalence of spontaneous preterm birth	Racial discrimination	Preterm birth	African American women, Latina women	Quantitative/ Cohort	Racial discrimination (2)	Preterm birth (7)	NA	Adjusting for multiple risk factors, African American and Latina women who experienced the highest tertile of discrimination had a higher prevalence of preterm birth compared with those who experienced discrimination less than once per year, adjusted hazard ratio (aHR) = 1.5 (0.7–3.1) and 3.6 (0.9–14.4), respectively
Garcia et al. (2019) [58]	To document racial/ethnic and nativity differences by gender in cognitive life expectancies among older adults in the United States.	Racial differences	Cognitive life expectancies	White, Black, Hispanic adults	Quantitative/ cross-sectional	Race (22), Age (24) 50 or more	Dementia (16)	Cognitive function score index (8)	Minority and foreign-born women are expected to spend a significantly lower proportion of their remaining years after age 50 in a cognitively normal state compared to White women.
Geronimus et al. (2020) [64]	To examine whether diverse residents of same neighborhoods exhibited different levels of allostatic load (AL) across race/ethnicity and poverty	Race/ethnicity, poverty level	Allostatic load	White, Black, and Mexican with age 15 and above	Community based participatory Quantitative/ Cross-sectional	Race/Ethnicity (22) and Poverty to Income Ratio (PIR) (60)	Allostatic load (17)	Everyday Unfair Treatment Scale (20), Negative Social Interaction Scale (47), Neighborhood Satisfaction Scale (48), Anger and Hopelessness Scale (3)	AL is statistically significantly associated with Poverty to Income Ratio (PIR) across the races
Groos et al. (2018) [32]	To summarize the ways in which researchers have quantified measures of structural racism for the purposes of empirical, quantitative investigation of its associations with physical and mental health outcomes.	Structural racism	Health inequity	People of color adults	Review/ Systematic review	Residential housing pattern (52), Socioeconomic status (53), criminal justice (51), immigration and border enforcement (52), home mortgage discrimination (53)	Health outcomes (1)	Generic survey index (28), Census Track based Redling Index (5), Census tract socioeconomic disadvantage index (7) and index of dissimilarity, Deferred Action for Childhood Arrivals (DACA) status (14), Dissimilarity Index, Felony Incarcerations (23), Institutional racism subscale of the Index of Race-Related Stress (IRRS) (34), Institutional racism subscale of the Index of Race-Related Stress-Brief Version (IRRS-B) (36), Isolation index, Racial bias in mortgage lending index and residential redlining index (53), Redlining index of mortgage discrimination (59)Relative Proportion Index (60), Self- Reported Scale (66)	Articles included measures of structural racism within the following domains, in order of frequency: residential neighborhood/housing, perceived racism in social institutions, socioeconomic status, criminal justice, immigration and border enforcement, political participation, and workplace environment.
Hall et al. (2015) [73]	To examine the relationships between health care professionals’ implicit attitudes about racial/ethnic groups and health care outcomes	Implicit bias, healthcare providers	Health outcome	Healthcare providers	Review/ Systematic review	Implicit bias of healthcare providers (50)	health care outcomes (18)	NA	Although some associations between implicit bias and health care outcomes were nonsignificant, results also showed that implicit bias was significantly related to patient–provider interactions, treatment decisions, treatment adherence, and patient health outcomes.
Hankerson et al. (2015) [78]	To explore socio-cultural factors that contribute to low treatment rates among depressed African American men in outpatient mental health care	Factors of treatment disparities	Depression	African American men	Review/ Narrative review	Cultural mistrust of healthcare providers (83) and misdiagnosis (84) and clinician bias (85)	Low treatment rate on depression (19)	NA	A complex array of socio-cultural factors, including racism and discrimination, cultural mistrust, misdiagnosis and clinician bias and use of informal support networks contribute to treatment disparities.
Hansen (2015) [30]	To examine associations between self-reported ethnic discrimination and health outcomes in the rural Sami population of Central and North Norway	Ethnic discrimination	Health outcome	Sami people	Quantitative/ Cross-sectional	Ethnic discrimination (2)	Waist circumference (20), blood pressure (21), total cholesterol (22), HDL cholesterol (23), triglycerides and glucose (24)	Dichotomized Scale (15)	For Sami people living in minority areas, self-reported ethnic discrimination is associated with all the negative health indicators; discrimination to be associated with several chronic conditions, such as chronic muscle pain, diabetes and metabolic syndrome.
Harnois et al. (2019) [69]	To assess the extent to which the everyday discrimination scale (EDS) produces estimates of perceived discrimination that are comparable across age, gender, education, and racial/ethnic-based groups	Everyday discrimination, education	Income	White, Black and Latinx adults	Quantitative/ Cross-sectional	Race (22)	Perceived discrimination (26)	Everyday Discrimination Scale (19)	Neither version of the scale generates estimates of discrimination that can be meaningfully compared across all racial/ethnic, age, gender, and education-based groups.
Heard-Garris et al. (2018) [74]	To summarize and discuss the current literature describing the associations between vicarious racism and child health to better inform practice and policy discussions in public health, medicine, and social science.	Vicarious Racism	Child health	African American Infant and Elementary school kids	Review / Systematic review	Perceived maternal discrimination/Perceived caregiver discrimination (86)	Infant health outcomes (Preterm birth (7), Cortisol reactivity (27), Birth weight (8)), Mental health (Depressive symptoms (10), Anxiety (28), Substance Use (29), Well-being (30), Depressive Symptoms (10)), Socioemotional health (Externalizing behavior (31), Internalizing behavior (32), Internalizing behavior Socioemotional difficulties (33), Self-esteem (34), Positive behavior(35)), Healthcare Utilization (Frequency of sick-child visits (36)), Physical Health (BMI (15), General Child illness (37), Weight-for-age (38)), Cognitive Development (Spatial ability (39)), Youth Health Outcomes (Depressive Symptoms (10))	Experiences of Discrimination (EOD) Scale, Everyday Discrimination Scale (EDS) (19), Measurement Instrument (43)	While all studies examined racism indirectly experienced by children, there was no standard definition of vicarious racism used.
Hearst et al. (2008) [62]	To examine whether residential segregation plays an independent role in high black infant mortality rates	Residential segregation	Infant mortality	Black women	Quantitative/ Cross-sectional	Residential Segregation (48)	Infant Mortality (4)	Isolation Index (37)	There were 1.12 excess infant deaths per 1,000 livebirths among black infants due to living in a segregated city compared with a nonsegregated city, although the difference was not statistically significant.
Kendall et al. (2020) [26]	To elucidate incarcerated Aboriginal women’s experiences of prison healthcare, investigate equity of access to culturally safe healthcare in prison, and identify pathways for improving the accessibility of culturally safe healthcare.	Targeted discrimination, intergenerational trauma	Access to healthcare	Incarcerated aboriginal women	Community collaborative participatory action research methodology/ Grounded theory	Race (22)	Access to healthcare (40)	NA	Aboriginal women experienced institutional racism and discrimination in the form of not being listened to, stereotyping, and inequitable healthcare compared with non-Indigenous women in prison and the community.
Kephart (2021) [72]	To summarize the relationship between racial residential segregation and greenness, provide an overview of the measures used, and suggest for practices for recontextualization	Racial residential segregation, access to green space	Health outcome	Black and white residents	Review/ Systematic review	Racial segregation (48), structural racism (2)	Green space in the neighborhood (41)	Dissimilarity Index (16)	Studies consistently demonstrate an association between racial residential segregation and less exposure to tree canopy coverage, vegetation, and parks. When residents of color do enjoy greater access to parks, these parks tend to be more congested and contain less amenities than parks located in areas with predominately White residents
Krieger (2012) [47]	To inform the methods of scientific study of discrimination and health	Discrimination	Health	White, Black, Asian, Hispanic, and Native American adults	Quantitative/ Narrative review	Wealth (59), Poverty (60), Unemployment (14), Incarceration of men (61), political parity ratio (62), No health insurance (47)	Infant mortality (4), Persons Year Lost (42), Mental Health (11)	Experiences of Discrimination (EOD) scale, Everyday Discrimination Scale (EDS) (19)	Socially patterned exposure-induced pathogenic pathways, mediated by physiology, behavior, and gene expression, that affect the development, growth, regulation, and death of our body’s biological systems, organs, and cells, culminating in disease, disability, and death.
Lane et al. (2004) [24]	to examine ecological-level risk factors leading to disparate rates in heterosexually transmitted HIV among women of color	Structural violence	HIV Infection Rates	Men and Women of color	Quantitative/ Cross-sectional	Disproportionate incarceration rates of African American men (61), residential segregation (48), gang turf (82), constraints on access to sexually transmitted diseases (STD) services (12)	HIV infection rates (43)	NA	The cumulative effect of the three pathways is to create a context that increases greatly African American women’s exposure to HIV and heightens the risk of its transmission. Rather than resulting from individual choices, this risk is mainly the result of the institutional and socio-structural patterns that result from the ecological model described
LaVeist (2003) [90]	To test the relationship between racial segregation and mortality using a multidimensional questionnaire-based measure of exposure to segregation	Racial segregation	Longevity	African American adults	Quantitative/ Cohort	Residential segregation (48)	Number of chronic condition (44)	Segregation index (65)	Respondents who were exposed to racial segregation were significantly less likely to survive the study period
Lo & Cheng, (2018) [60]	To measure minority individual's social status factors and frequency of discrimination experiences and impact on mental health	Racism, social status	Mental health	Asians, Latinx and African American adult	Quantitative/ Cross-sectional	Immigration status (76), gender (23), marital status (10), education (11), Income to needs ratio (76)	Mental health (11)	Frequency of Discrimination Experience Index (26)	Across races better mental health was associated with male, gender, higher income, marriage, more education, and less frequent discrimination experiences among blacks.
Malcome (2021) [91]	To examine difference in symptoms of depression and its relation with age and community experience	Racial discrimination, sense of community belonging	Depression	Black mothers	Mixed Method/ Longitudinal randomized controlled trial	Community Violence (4), Racism (2)	Depression (10)	Community violence scale (10), Beck Depression Inventory Scale (4), racial discrimination scale (54), Community Belonging scale (9)	Exposure to community violence, fear of violence victimization, structural racism negatively affects the mental health of low-income black mothers
Martin et al. (2019) [25]	To document lived experience of dislocation, poor health, and homelessness of western Australian aboriginal people	Homelessness and housing	Health and wellness	Aboriginal people	Community based Participatory Qualitative/ Exploratory qualitative	Homelessness (1)	Health and wellbeing (30)	NA	Participants experienced disconnection from kin and country, are likely to have serious risk to personal safety, homelessness, and problematic health due to colonization, dispossession, and racism.
Mehra (2020) [92]	To determine the extent to which structural stigma is associated with racial disparities in adverse birth outcomes	Structural stigma, interpersonal stigma	Birth outcomes	Black, White and Hispanic mothers with preterm and low birth weight infant	Mixed Method/ Systematic review & meta-analysis	Residential segregation (48)	Preterm birth (7) and Low birth weight (8)	Five dimensions of segregation	Among the black mothers, exposure and hypersegregation were associated with increased risk of multiple adverse birth outcomes and greater black-white and white-Hispanic disparities in preterm birth in racially isolated counties.
Mobley et al. (2017) [93]	To assess the association between a measure of social cohesion/support (residential segregation) and health outcomes (late-stage colorectal cancer stage (CRS) diagnosis)	Residential segregation	likelihood of late-stage CRC diagnosis	Persons with CRC diagnosis	Quantitative/ Non-experimental survey	Residential segregation (48)	Late-stage CRC diagnosis (45)	Isolation Index	Living in highly segregated Asian communities is highly associated with higher likelihood of late CRC diagnosis.
Neblett Jr (2019) [77]	To discuss three pressing challenges in the study of racism as a social determinant of health and identifies ideas to guidefuture psychological and behavioral research	Racism	Racial health inequity	People of color adults	Review/ Narrative review	Status-related stressors (e.g., sexism heterosexism (64), religious discrimination (65), disability discrimination (66), ageism (22), classism (67), and sociocultural variables (e.g., worldview (68), spirituality (69), racial/ethnic identity (22), acculturation (70)	Psychological (e.g., anger (46), fear (47)) and physiological (e.g., immune (48), neuroendocrine (49), and cardiovascular (50)) stress responses	Index of Race-Related Stress (IRRS) (34)	Institutional, cultural, and structural racism, the incorporation of developmental health and resilience perspectives, the use of diverse methods and transdisciplinary approaches, and improved education and training should be considered.
Nikolova et al. (2015) [68]	To identify factors that predict the circumstances people with disabilities face, including poverty.	Disability, geo-social disparity	Health outcome	People with disability	Quantitative/ Cross-sectional	Household characteristics and conditions (46), education (11), employment (14) and health coverage (47)	Disability pattern (51)	The Global Moran Index (I) (30) and The Local Moran's Index (LISA) (41), Akaika Information Criteria (AICc) (2)	People with disabilities living below the poverty line experience high segregation levels in the semi central zones of Dallas. In Monterrey, people with disabilities clustered in central areas of the city. High goodness of fit (R > 0.8 for Dallas data and R > 0.7 for Monterrey data, respectively and predictability of disability prevalence when social disadvantage factors such as unemployment, housing insecurity, household living conditions, and lack of education were present.
Nyika & Murray-Orr (2017) [29]	To examine the importance of critical race theory (CRT) - social constructivist hybrid approach in race research	Health promoting schools (HPS)	Constructivism	African immigrant students	Qualitative/ Narrative review	Race (22), Gender (23), Culture (25), Language (31) and Legal System (32),	Health Outcome (1)	NA	CRT and social constructivist approach can be integrated in HPS framework
Pabayo et al. (2019) [61]	To determine whether indicators of structural racism are associated with the individual odds for infant mortality among white and black infants in the US.	Structural racism	Infant mortality	Black and white mothers	Quantitative/ Cross-sectional	prison incarceration (77) and juvenile custody rates (78); sentencing rates (79) and capital punishment (80); educational attainment (11) (proportion of population aged 25+ with bachelor’s degree or higher); unemployment (14) (proportion of civilian labor force not currently employed); professional occupational status (81) (proportion employed in management, business, science, and arts occupations); and median household income (74)	Neonatal and Infant mortality (4)	Index of concentration at the extremes (ICE) (11)	Compared to the lowest tertile ratio of relative proportions of blacks to whites with a bachelor’s degree or higher— indicative of low structural racism—black infants, but not whites, in states with moderate (OR = 1.12, 95% CI = 0.94, 1.32) and high tertiles (OR = 1.25, 95% CI = 1.03, 1.51) had higher odds of infant mortality
Palacio et al. (2020) [94]	To create a weighed Social Determinants of Health (SODH) score and to test the impact of each SDOH factor on the Framingham risk score (FRS) and on individual traditional CVD risk factors	Social determinants of health (SODH)	Cardiovascular Disease	All patients	Quantitative/ Retrospective cohort	SODH Exposure (75)	10-year Framingham risk score (FRS) for CVD (51)	Framingham risk score (FRS) (25)	An increasing SDOH score correlated with being a member of a racial/ethnic minority group, not being employed, having an education of high school or less, residing in a community with lower household income according to Census data, and having a higher prevalence of baseline CVD risk factors (P < .01); Increasing quartile of SDOH score was significantly associated with higher systolic blood pressure, FRS, glycated hemoglobin, and smoking pack-years (P < .05)
Paradies et al. (2015) [2]	To review the literature focusing on the relationship between reported racism and mental and physical health outcomes	Racism	Mental and Physical health outcomes	Adults of people of color	Review/Systematic review	Reported racism (58)	Mental health (11), Physical Health (52) and General Health (53)	Schedule of Racist Events (SRE) (64), Racism and Life Experience Scales (RaLES) (57), Experiences of Discrimination (EOD), Perceived Racism Scale (PRS), Everyday Discrimination Scale (EDS) (19), Perceived Ethnic Discrimination Questionnaire (PEDQ) (50), Multidimensional Inventory of Black Identity (MIBI): public regard subscale (45), Nadanolitization scale (46)	Racism was associated with poorer mental health (negative mental health: r = -.23, 95% CI [-.24,-.21], k = 227; positive mental health: r = -.13, 95% CI [-.16,-.10], k = 113), including depression, anxiety, psychological stress and various other outcomes. Racism was also associated with poorer general health (r = -.13 (95% CI [-.18,-.09], k = 30), and poorer physical health (r = -.09, 95% CI [-.12,-.06], k = 50).
Paradies et al. (2014) [27]	To systematically review and appraise evidence of healthcare provider racism and assess current approaches to measuring racism amongst healthcare providers	Interpersonal Racism	Healthcare inequity	Healthcare providers	Quantitative/ Systematic review	Race of healthcare providers (3)	Quality of healthcare (54)	Social distance scale (68), Affective racial attitude (1), Contemporary racism awareness scale (12), Ethnic attitude scale (18), Feelings of warmth scale (22), Implicit Association Test-IA (32), Knowledge and attitude towards immigrants scale (38), Multicultural counseling knowledge and awareness scale (44), New racism scale (49), Racial preference scale (55), Scale on Beliefs about race related policies (61), scale on race-based meritocracy (62), Scale on self-perception on racism among providers (63), Semantic differential situational attitude scale (67), Vignettes (70), Visible Racial (71), Ethnic identity attitude scale (72)White racial identity attitude scale	Statistically significant evidence of racist beliefs, emotions, or practices among healthcare providers in relation to minority groups was evident in 26 of these studies. Although a number of measurement approaches were utilized, a limited range of constructs was assessed.
Prasannan et al. (2021) [59]	To determine how social determinants of health are associated with severe acute respiratory syndrome and severity of coronavirus illness	Social determinants of health	acute respiratory coronavirus syndrome	Pregnant women	Quantitative/ Cross-sectional	Household income (74), unemployment (14) and high school education (11)	Acute respiratory syndrome (55)	Zone Improvement Plan (ZIP) (73)	Pregnant patients who had a positive test result were more likely to be younger or higher parity, belong to minorized racial and ethnic groups and reside in low-income neighborhoods with less educational attainment. Obesity, income and education were associated with coronavirus disease 2019 severity.
Prioleau (2021) [95]	To explore intersectional experiences of black women in relation to gendered-racism, race-related stress, socioeconomic status (SES), and its impacts on total wellness factors	Gendered racism, race related stress and socioeconomic status	Health Wellness	Black women	Quantitative/ Non-experimental survey	Gendered racism (23), race-related stress (22), socioeconomic status (SES) 53,	Wellness score (56)	Gendered racial microaggression scale (27), Index of race related stress-brief and five factor wellness inventory (35)	More gendered racial microaggression on certain domains were associated with higher wellness scores. Higher scores on race related stress and the lowest SES status group score were associated with lower overall wellness scores.
Pursch et al. (2020) [31]	To explore the provision of health services to migrants in Calais and La Linière, through a structural violence lens	Structural violence	Provisions of health services	NGO professionals	Qualitative/ Exploratory qualitative	Structural violence (2)	Access to healthcare (40)	NA	Structural realities including violence appeared to negatively affect migrant social determinants of health, reducing healthcare access, social inclusion, and sense of empowerment.
Ricks et al. (2021) [75]	To explore what research methods are being used to ascertain the training healthcare workers are receiving post-licensure and to identify the goals and outcomes of this training	Racism, implicit bias	Racial health inequity	Healthcare providers	Review/ Systematic review	Race of healthcare providers (3)	Increased self-awareness (57), Racial attitudes (58), Knowledge attainment (59), Self-reported skills like decision-making (60), Number of PEH receiving COVID test (61), Length of stay (62)	NA	Reported outcomes included increased self-awareness of implicit bias.
Ryus et al. (2021) [96]	To examine the utility of community based participatory research approach (CBPR) to address structural racism	People experiencing homelessness (PEH)		Homeless people	Qualitative/ Exploratory qualitative	Homelessness (1)	Number of PEH receiving COVID test in ED (61), Length of Stay (62)	NA	Community based participatory research approach (CBPR) was found to be effective in designing need addressing interventions for PEH.
Singer et al. (2021) [82]	To examine the association between student, school and neighborhood factors with chronic absenteeism	Student, school and neighborhood factors	Chronic absenteeism	People of color K to 12 grade students	Quantitative/ Ecological	Math and English Test Score (5), Violent Crime Rate (6), Residential Vacancy rate (7), School stability rate (8), Asthma rate (9)	Chronic absenteeism (63)	Index of macro-level factors (33)	Ecological factors were significantly associated with chronic absenteeism
Tester et al. (2010) [79]	Is the term “structural violence” appropriate to describe the health outcomes of the housing provided to Inuit in the 1950s and 1960s?	Structural violence	TB Outbreak	Eskimo	Review/ Case	Poor housing (52)	TB outbreak (64)	NA	While prior viral epidemics were relevant, living conditions at Eskimo Point contributed significantly to development and spread of the disease.
VanPuymbrouck et al. (2020) [66]	To explore disability attitudes of health care providers	People with disability (PID), bias	Access to quality healthcare	Healthcare providers	Quantitative/ Cross-sectional	Disability (44)	Providers' attitude (65)	Likert Scale (40)	Despite majority of providers self-reporting not being biased against people with disabilities, implicitly, the overwhelming majority were biased.
White et al. (2012) [76]	To develop a conceptual framework for investigating the role of racial/ethnic residential segregation on health care disparities.	Racial/ethnic residential segregation	Healthcare disparities	Healthcare providers	Review/ Narrative review	Geographical segregation (26) and Health facility-based segregation (43)	Health inequity (66)	Five segregation scale (24)	Racial/ethnic residential segregation is a key factor driving place-based healthcare inequities.
Williams et al. (2019) [10]	To review the evidence linking primary domains of racism to mental and physical health outcomes	Structural racism, Cultural racism & individual level discrimination	mental and physical health outcomes	African American adults	Qualitative/ Narrative review	Residential segregation (48), ideology of inferiority in the values (71), language (72), symbols (73), unstated assumptions (74), discrimination (2)	Health outcomes (1)	NA	Segregation was associated with increased risk of low birth weight and preterm birth for blacks. Individual level unconscious bias is associated with inferior medical care for minor ethnic groups. Self-reported discrimination is associated with negative health outcomes.

## Discussion

Our review synthesized the growing and recent body of literature on structural racism and highlighted the current methods of structural racism research. Our review particularly offers a comprehensive synthesis of methodological current practices and issues in terms of study design, inventory of measures of exposure of structural racism and health outcomes, inventory of comprehensive list of scales/indexes of measurement and current methodological challenges. This review highlights several important findings:

A striking finding was that the current structural racism research is heavily based on quantitative research approach followed by mixed method and qualitative research. Several studies used the community based participatory research approach which was found to be effective. Similar suggestions on using mixed method research approach have also been given in recent other studies [20, 80]. It has been argued that the use of mixed method (supplementing quantitative data with qualitative data in sequential design or triangulation approach) can facilitate a greater understanding of the social determinant of health by describing and analyzing multidimensional and multiple impact pathways of health outcomes of structural racism [20]. This calls for the need of more research on the use of qualitative approach in studying structural racism.

Secondly, this review shows that the majority of the current racial research employs cross-sectional study design suggesting the dearth of longitudinal studies that consider multiple impact pathways and dimensions. It also indicates the paucity of longitudinal studies in the current research trend and practices by racism scholars. Furthermore, this review also highlights the limitations associated with the cross-sectional design. Several longitudinal studies found that cross-sectional designs were associated with type II error and biases in relation to physical outcomes of structural discrimination [91]. In another study in the United States cross-sectional design was found with the limitation to make conclusion about the temporality in the association between the exposure of discrimination and anxiety scores [97]. Similar limitations on the examination of temporality and causal assumptions have also been reported in study with indigenous South Australians [39]. Our review highlights the several gaps regarding the appropriate study designs: 1) there is no agreed upon best practices in selecting study design while studying structural racism in a given country context, 2) there is critical need to conduct more research with particular focus on the suitability of different study design and develop a gold standard for structural racism research, and finally 3) there is need for methodological innovations for better understanding which can inform the design of future programs, policy or practices regarding structural racism.

Some other key methodological challenge are the unavailability of data on structural levels [10] and variation of estimates by the geographic unit of analysis which has been documented in several other studies [9, 40, 41]. When residential segregation is considered as exposure, the association between segregation and health outcome tend to vary as the most reliable estimates are found for smaller unit of analysis. This analysis is not consistent for all health outcomes. It often becomes impossible to differentiate the potential mediating and moderating effects in the association. The current research practice adopted by some scholars to overcome this challenge is to control variables related to socio-economic condition which are the pathways of how racism affects health outcomes [40, 41]. This sheds light on future research needs to identify the proximal mechanisms, interaction pattern between the exposure and health outcomes by using longitudinal data and advanced statistical method to develop concrete understanding around temporality and causality.

Thirdly, our review, to our best knowledge is one of the first reviews to systematically synthesize all available and reported measures of exposure and health outcomes in relation with structural racism as social determinants of health. Our review has documented a total of 87 measures of exposure and 56 measures of health outcomes. It also highlights the most common clusters of exposures which include educational attainment, employment and income, sociodemographic characteristics. The most common clusters of health outcomes found in our review were infant health outcome, chronic condition, mental health, and quality of life. Similar observations have consistently been documented in several studies [8, 95]. Such as a wide range of clusters of exposures and outcomes pinpoints the magnitude of public health implications in the United States and other high-income countries. This also suggests that there is critical need to develop a comprehensive and integrated framework for measures of exposure and outcomes related to the study of structural racism.

Fourthly, our review documents all available scales or indexes of measurement related to structural racism available in the literature. It lists 73 scales of measurement and discusses methodological challenges related to widely used scales of measurement such as individual focus, self-reporting and personally mediated, non-linear nature which are associated with bias and confounding. This have been documented in other studies [85].

The other limitations with the current indexes of measurement are more focus on liner domain-based measurements, failure to study multidimensional and multilevel impacts of structural racism, and applicability to limited number of ethnic groups. These findings are consistent with recently published literature [13]. Our review demonstrates that there is no scientific consensus on the use of index of measurements that help us further understand and explain the dynamics and pathways of multilevel interactions of mutually reinforced systems and institutions.

Finally, our review clearly highlights the gaps in the current research on structural racism. Some of the widely documented gaps are lack of systematic, longitudinal studies that examine multiple pathways and ecological factors by which racism can affect health over the life course [60], the use of single dimensions of structural racism (e.g., housing, education, employment, incarceration, etc.) [20], exclusion of the appropriate respondents in the appropriate settings (e.g. exclusion of prisoners in the most of the studies) [33], methodological challenges involving individuals, levels and spatiotemporal scale [33]. These findings are consistent with the findings of other studies [91, 95]. Last not but the least, our review shows the lack of best practice regarding the selection of measures of exposure and outcome, study design and index of measurement and therefore, calls for more research initiatives and develop a standard guideline for the researchers interested in structural racism. Such a call has also been made in other study [93]. The findings from our review can guide researchers, academicians, and other relevant stakeholders in designing future research and programs on structural racism.

### Limitations

This scoping review anticipates several limitations and biases. One bias could be the selection bias as we included studies which were published in English. Secondly, although comprehensive searches across databases were done, it is possible to miss relevant sources which could have been eligible for this review. Thirdly, the review did not conduct quality assessment of the included studies. Finally, we found that more studies on structural racism were done in the United States than other countries. We therefore recommend the exercise of caution in the use of findings in other context. Despite these limitations our review has documented the current research trend, practices, challenges, and future research needs on structural racism.

### Conclusion

Our scoping review found that despite repeated calls from racism scholars for more comprehensive approaches, traditional research methods are being followed by most of the scholars. We found that there is a severe lack of longitudinal studies and availability of structural or ecological data. There is growing understanding among the racism scholars that it is imperative to understand the ways in which the social or ecological context including all the structure, institutions, laws, policies, and practices affect the health of a racial group. It has also been recognized that a detailed and comprehensive characterization of the exposures in their social context is required [98, 99]. Our review sheds lights on several key gaps and research priorities on structural racism. First of all, there is a need to develop agreed upon measures with concrete indicators that can comprehensively assess multidimensional and multilevel health outcomes of structural racism. Secondly, the current methodological gaps such as lack of consensus or framework on appropriate study design that can capture the complex interactions of systems and interconnected institutions need to be addressed through further research on structural racism. Thirdly, there is also a need to develop a framework on choosing the index of measurement which needs to be selected prior to the design of research. We, therefore, recommend the development and use of new structural racism measures which could be a good fit at different levels and geographical location for consistent and reliable estimates. We also recommend the use index measures based on a set of concrete indicators which capture complex interactions of exposure and outcomes and undertaking of longitudinal studies using a life-course approach to measurement.

Finally, we acknowledge that there is also growing recognition among the racism scholars that studying structural racism requires more scientific, rigorous and context specific study methods of structural racism or discrimination.

## Supplementary Information

**Additional file 1.**

## Data Availability

"The dataset(s) supporting the conclusions of this article is(are) included within the article (and its additional file(s)).

## Abbreviations

AIDS: Acquired Immunodeficiency Syndrome
BRFSS: Behavioral Risk Factor Surveillance System
CINAHL: Cumulative Index to Nursing and Allied Health Literature
DACA: Deferred Action for Childhood Arrivals
EDS: Everyday Discrimination Scale
EDS: Experience of Discrimination Scale
EOD: Experiences of Discrimination
FRS: Framingham Risk Score
HIV: Human Immunodeficiency Virus
ICERD: International Convention on the Elimination of All Forms of Racial Discrimination
IRRS: Index of Race Related Stress
IRRS-B: Index of Race-Related Stress-Brief Version
LISA: Local Moran's Index
MLD: Major Life Discrimination
MIBI: Multidimensional Inventory of Black Identity
PEDQ: Perceived Ethnic Discrimination Questionnaire
PRS: Perceived Racism Scale
PoRS: Perceptions on Racism Scale
RaLES-B: Racism and Life Experience Scale—Brief Version
RaLES: Racism and Life Experience Scales
RRS: Racism Reaction Scale
SODH: Social Determinant of Health
STD: Sexually Transmitted Diseases
SRE: Schedule of Racist Life Events
ZIP: Zone Improvement Plan
